# Selection on the morphology–physiology‐performance nexus: Lessons from freshwater stickleback morphs

**DOI:** 10.1002/ece3.3644

**Published:** 2017-12-22

**Authors:** Sergey Morozov, Tuomas Leinonen, Juha Merilä, R. J. Scott McCairns

**Affiliations:** ^1^ Ecological Genetics Research Unit University of Helsinki Helsinki Finland; ^2^ Department of Biosciences University of Helsinki Helsinki Finland; ^3^ ESE, Ecology and Ecosystem Health INRA Rennes France

**Keywords:** ecophysiology, *Gasterosteus aculeatus*, metabolic rate, morphotype, physiological adaptation, respirometry, selection, swimming performance

## Abstract

Conspecifics inhabiting divergent environments frequently differ in morphology, physiology, and performance, but the interrelationships amongst traits and with Darwinian fitness remains poorly understood. We investigated population differentiation in morphology, metabolic rate, and swimming performance in three‐spined sticklebacks (*Gasterosteus aculeatus* L.), contrasting a marine/ancestral population with two distinct freshwater morphotypes derived from it: the “typical” low‐plated morph, and a unique “small‐plated” morph. We test the hypothesis that similar to plate loss in other freshwater populations, reduction in lateral plate size also evolved in response to selection. Additionally, we test how morphology, physiology, and performance have evolved in concert as a response to differences in selection between marine and freshwater environments. We raised pure‐bred second‐generation fish originating from three populations and quantified their lateral plate coverage, burst‐ and critical swimming speeds, as well as standard and active metabolic rates. Using a multivariate *Q*_ST_‐*F*_ST_ framework, we detected signals of directional selection on metabolic physiology and lateral plate coverage, notably demonstrating that selection is responsible for the reduction in lateral plate coverage in a small‐plated stickleback population. We also uncovered signals of multivariate selection amongst all bivariate trait combinations except the two metrics of swimming performance. Divergence between the freshwater and marine populations exceeded neutral expectation in morphology and in most physiological and performance traits, indicating that adaptation to freshwater habitats has occurred, but through different combinations of traits in different populations. These results highlight both the complex interplay between morphology, physiology and performance in local adaptation, and a framework for their investigation.

## 1 INTRODUCTION

Adaptation to novel environments can involve changes at multiple phenotypic levels. Perhaps due to the relative ease at which they are observed and measured, changes in morphological traits have featured prominently in studies of adaptation to novel habitats (Gavrilets & Losos, 2009; Reznick & Ghalambor, 2001; Rundell & Price, 2009). However, morphological similarity may belie more complex physiological diversity (Lindholm, 2014). Conversely, physiological constraints may also limit adaptation (Ricklefs & Wikelski, 2002), with the corollary to this being that selection on physiological processes must be an equally important aspect of adaptation to new habitats. Indeed results from genome scans increasingly reveal signals of selection acting on physiological processes (Akey et al., 2010; Gautier et al., 2009; Shimada, Shikano, & Merilä, 2011; Simonson et al., 2010), and on metabolism in particular (reviewed in Marden, 2013). While these types of studies can provide initial evidence in support of adaptive divergence, the case of the Andean Sparrow (*Zonotrichia capensis*) highlights their potential limitations: A genome scan suggested selection acting on metabolic pathways, but this result was contradicted by experimental tests of associated enzyme affinity (Cheviron et al., 2014). Thus, direct measures of physiological processes are integral in the context of studying putative adaptive divergence. Likewise, neither morphology nor physiology can be considered independent of their relationships with performance and their interactive effects on Darwinian fitness (Arnold, 1983). Yet, despite its immediate links to fitness, there has been little evidence that selection is necessarily stronger on performance traits than on morphology (Irschick, Meyers, Husak, & Le Galliard, 2008). Moreover, studies on how fitness mediates the relationship between performance and physiology have been identified as particularly lacking (Careau & Garland, 2012). An integrated study of whether and how selection has shaped differentiation of morphological, physiological, and performance traits amongst conspecifics adapted to divergent environments provides one way of tackling this lacuna (Irschick & Garland, 2001).

Metabolism may be the physiological process most relevant to such an endeavor, ultimately governing the amount of energy available for partitioning to growth, predator avoidance, reproductive investment, and most other components of fitness (Fry, 1971). Indeed, it is argued that energetics plays a central role in mediating the physiology–morphology–performance–fitness paradigm (Arnold, 1983; Careau & Garland, 2012); thus, metabolic rate may be viewed as a parameter of fundamental importance. As many factors both intrinsic and extrinsic to the individual have an influence on metabolic rate, measurements are typically divided between resting/basal and active states. Resting metabolic rate—typically referred to as standard metabolic rate (SMR) in ectothermic species, such as fishes (Chabot, Steffensen, & Farrell, 2016)—is the minimal level of oxygen consumption required to maintain basic biological functions of a fasting animal (Priede, 1985). SMR varies considerably among individuals and populations, and correlates with a number of fitness‐related traits; however, fitness associations are also modulated in a context‐dependent manner with varying environmental conditions (reviewed in Burton, Killen, Armstrong, & Metcalfe, 2011). Active metabolic rate (AMR) defines the upper limit of oxygen consumption during maximum sustained/prolonged aerobic activity (Fry, 1971; Pitcher & Hart, 1983). Although studies of metabolic rate have been increasing in evolutionary biology, particularly with respect to individual performance and the potential fitness consequences of its variability (Metcalfe, Van Leeuwen, & Killen, 2016), few have documented the relative contributions of selection versus drift underlying differentiation in metabolic traits amongst conspecifics from ecologically divergent habitats.

Another important set of physiologically linked traits contributing to Darwinian fitness of aquatic organisms are different aspects of swimming performance (Reidy, Kerr, & Nelson, 2000). Prolonged swimming speed—the speed which can be maintained from 20 s up to 200 min, typically ending in fatigue (Beamish, 1978)—is positively correlated with maximum oxygen consumption (Brett, 1964; Dalziel, Vines, & Schulte, 2012), male territoriality (Kolok, 1999), migration capacity (Tudorache, Blust, & De Boeck, 2007), and routine activity (Fuiman & Webb, 1988). Burst swimming speed—the maximum attainable speed, typically maintainable for periods <20 s (Beamish, 1978)—can impact feeding efficiency and predator avoidance (McGuigan, Franklin, Moritz, & Blows, 2003). Moreover, both prolonged and burst swimming speeds are also strongly associated with morphological traits such as caudal fin size, body shape, and defensive armoring (Bergstrom, 2002; Taylor & McPhail, 1986; Webb, 1982). Given its complex interactions with metabolism and morphology, swimming performance is a likely target for functional trade‐offs and/or correlational selection.

**Figure 1:**
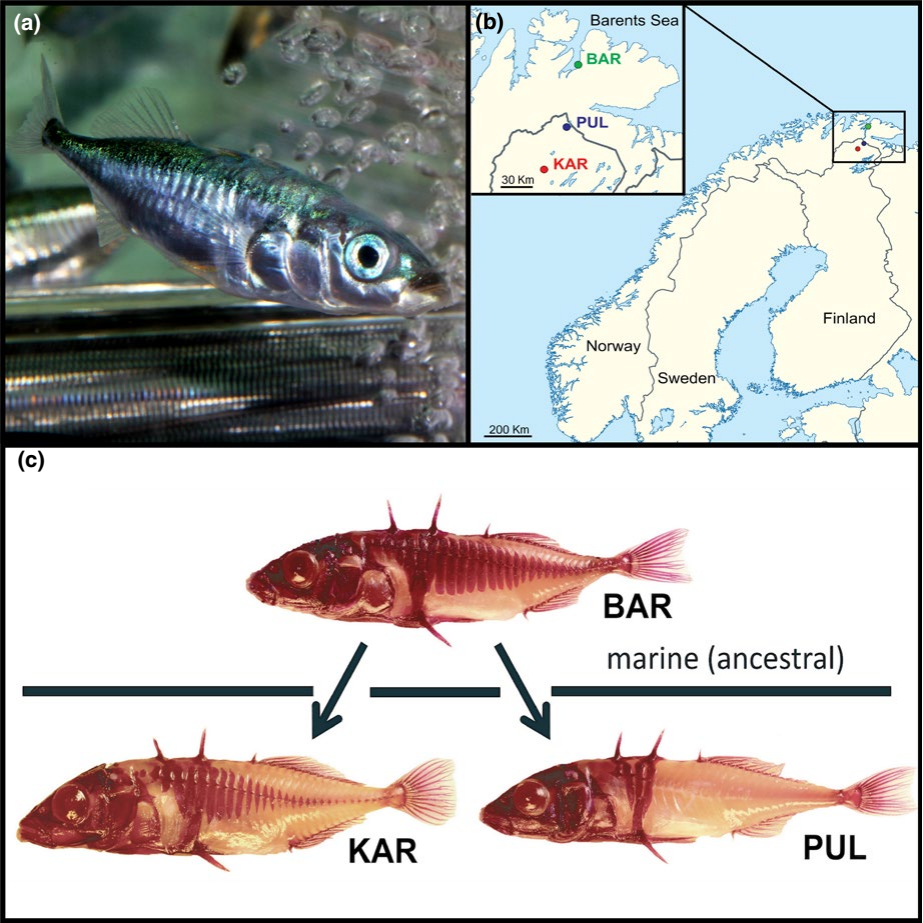
(a) The study species, three‐spined stickleback (*Gasterosteus aculeatus* L.) Copyright SM. (b) Location of reference populations. (c) Morphotypes found in the reference populations. The “ancestral” population originates from the Barents Sea (BAR), comprising full‐plated marine fish. Two allopatric freshwater morphotypes can be found in Finnish Lapland: the novel “small‐plated” morphotype, sampled from Lake Karilampi (KAR), and the typical low‐plated form sampled from Lake Pulmankijärvi (PUL)

The three‐spined stickleback (*Gasterosteus aculeatus* L., Figure 1a) represents an ideal model for studies in evolutionary physiology and morphology. A marine fish in origin, postglacial colonization of freshwater habitats has resulted in dramatic phenotypic responses to novel ecological pressures (Bell & Foster, 1994). One of the most conspicuous involves reduction in the number of lateral plates, large modified scales that cover the posterior body, and offer protection from predatory fishes (Reimchen, 1983, 2000). While marine populations are composed mostly of full‐plated individuals (but see Münzing, 1963; Lucek, Roy, Bezault, Sivasundar, & Seehausen, 2010), freshwater populations are frequently dominated by a “low‐plated” phenotype that has lost its posterior lateral plates (Bell & Foster, 1994; Colosimo et al., 2005; Jones et al., 2012), most likely in response to selection pressures exerted by altered predation regimes and habitat structure in the new environment (Barrett, 2010; Leinonen, Herczeg, Cano, & Merilä, 2011; Reimchen, 1992). Although the “low‐plated” morph is the typical form found in freshwaters throughout the species, global distribution (Colosimo et al., 2005; Hagen & Gilbertson, 1972; Münzing, 1963), reduced lateral plate number is not the only way by which posterior lateral plate coverage is reduced. An alternative pathway to decreased lateral plate coverage can be seen in a “small‐plated” freshwater morph found in several ponds in Finnish Lapland (Leinonen, McCairns, Herczeg, & Merilä, 2012) and in streams of Lake Constance (Marques et al., 2016). Although these populations possess a full set of lateral plates, the size/height of lateral plates has been dramatically reduced relative to the ancestral state (Leinonen et al., 2012). In contrast to the low‐plate morphotype, it is unknown if reduction in plate size is the result of selective or neutral evolution.

Adaptation to the freshwater environment also appears to have been accompanied with differentiation on a physiological level, and in performance traits. In addition to divergence in osmoregulatory physiology (Taugbøl, Arntsen, Østbye, & Vøllestad, 2014; Wang et al., 2014), low‐plated freshwater forms may differ from the ancestral full‐plated morph in both swimming performance and metabolic capacity (Dalziel, Vines et al., 2012; Tudorache et al., 2007). Whether these changes are the result of stochastic processes, direct selection on physiological traits, or correlational selection somewhere within the nexus of morphology and physiology remains an open question. What is certain is that elucidating the evolutionary mechanisms underlying physiological differentiation among marine and freshwater morphotypes of sticklebacks are required not only for a deeper understanding of this model system, but also for our understanding of whole‐organism adaptation in general (Arnold, 1983; Careau & Garland, 2012).

The principal aim of this research is to test whether differentiation of two freshwater morphs from their ancestral marine population occurred via neutral or selective evolution. We focus on traits whose divergence in freshwater has been putatively linked to fitness (e.g., lateral plates, burst swimming speed) and formally test for signatures of selection. Notably, we take a multivariate approach to determine if divergence may be due to direct or correlated responses to selection, and to test for correlational selection between physiological and functional traits.

## 2 MATERIALS AND METHODS

### 2.1 Fish collection, breeding and rearing

Broodstock were collected from three discrete populations in northern Fennoscandia during the spawning season of 2011 (Figure 1b). This included two populations of freshwater sticklebacks, each with a distinct morphotype (Figure 1c): the typical low‐plated form from Pulmankijärvi (PUL), and the small‐plated morphotype from Karilampi (KAR). The third population comprised full‐plated marine fish collected from the Barents Sea, the ancestral/source population of the freshwater populations (Mäkinen, Cano, & Merilä, 2006; Mäkinen & Merilä, 2008). Wild‐caught fish were used to establish 40–60 half‐sib families for each population following protocols for in vitro fertilization (Barber & Arnott, 2000). Pure‐bred first‐generation (*F*_1_) fish were raised under common environmental conditions for one year (freshwater; 16°C ± 2°C; 16 hr:8 hr light:dark photoperiod; ad libitum feeding with chironomid larvae), followed by 2 months of simulated overwintering (4°C; 24 hr darkness; reduced feeding). Sexual maturity was induced by gradually restoring temperature to 16°C (1°C per day) and increasing photoperiod to 24 hr light (natural light regime in study populations during the breeding season). When females were fully gravid and males displayed nuptial coloration, individuals from unrelated families were selected at random to produce pure‐bred second‐generation (*F*_2_) families.

Experimental fish originated from eight full‐sib families within each population (24 *F*_2_ families in total). Fish were reared in family groups consisting of 15 individuals. Families were kept in 6 L tanks on a stand‐alone, recirculating aquatic housing unit (Aquaneering Inc., San Diego, CA, USA) and maintained under rearing conditions described in the preceding paragraph. At one‐year post‐hatch, family groups were reduced to five individuals (10 individuals selected at random for a companion study), which were used for experimentation (*N* = 120; *n* = 40 per morph). All fish used for experimentation were the same age and developmental stage (i.e., sexually immature adults). Mean body size did not differ among groups (Fig. S1a), and each morphotype exhibited a common length–weight trajectory (i.e., no significant difference in scaling exponents; *F*_[2]_ = 2.58, *p *=* *.081); however, length‐specific mass did differ among groups (Fig. S1b; *F*_[2]_ = 12.05, *p *<* *.001).

### 2.2 Swimming trials

#### 2.2.1 Burst swimming

Fish were placed individually in a 20 cm long (ca. 5–6 body lengths; 1–2 body lengths width) observation chamber marked with distance graduations, and startled to elicit burst swimming in response to simulated predator strikes by thrusting a pair of blunt forceps toward the resting fish (similar to Bergstrom, 2002), or in the case of unresponsive fish, by pinching the caudal fin (Redpath et al., 2010). A minimum of three separate escape attempts were filmed with a digital video camera (50 fps) mounted above the chamber. Following swimming trials, fish were placed into individual 1 L tanks on the recirculating system and allowed to recover for a minimum of 48 hr prior to respirometry.

To measure swimming speed, videos were advanced to a frame in which fish had adopted the “C‐bend” which precedes a rapid burst of movement (Beamish, 1978), and tracked over the course of 5–10 frames (0.1–0.2 s). Instantaneous velocity was measured between each frame using the Tracker 4.82 video analysis software (Brown, 2014). Burst swimming speed (*U*_burst_) was estimated as the maximum velocity recorded in three trials.

#### 2.2.2 Critical swimming

Maximum prolonged swimming performance was estimated using the standard critical swimming speed (*U*_crit_) protocol (Brett, 1964). The measurements were conducted in 250 ml Blazka‐type swim tunnels (Loligo Systems, Denmark). Fish were forced to swim against current with the velocity of water flow gradually increased by 2.5 cm/s every 10 min until fish exhibited a transition from aerobic to anaerobic respiration, indicated by a rapid change in swimming gait. *U*_crit_ was calculated using Brett's (1964) equation:

(1) $$U_{\mathrm{crit}}=U_{f}+U_{i}\times t/t_{i},$$

where *U*_f_ (cm/s) is the water velocity maintained for a full‐time interval prior to the fatigue velocity, *U*_i_ is the increment velocity (here 2.5 cm/s), *t*_i_ is the prescribed time interval (min), and *t* is the amount of time (min) spent at the fatigue velocity. Following prolonged swimming performance testing, fish were allowed to rest for approximately 2–3 hr, during which time tunnels were maintained at open flow (5 cm/s velocity).

### 2.3 Respirometry

Four swim tunnels connected to a four‐channel respirometry system (DAQ‐PAC‐F4; Loligo Systems, Denmark) were used to measure standard (SMR) and active (AMR) metabolic rates. Each swim tunnel was located inside an 8‐L chamber on a recirculating system maintained at a constant temperature of 17 ± 0.5°C and ≥ 8.5 mg O_2_/L. Respirometry measurement consisted of several steps conducted over a ca. 24‐hr period. These began in the evening with the cleaning of oxygen sensors and pretest measurements of oxygen consumption due to microorganisms in the system (background respiration). Resting (i.e., standard) metabolic rate (SMR) was measured in the absence of stimuli following a period of acclimation to the respirometry system. The next morning, maximum respiratory capacity was determined by first measuring the critical swimming speed (*U*_crit_) of each fish to define water velocities for the subsequent measurement of active metabolic rate (AMR). A final estimate of background respiration was then taken for each swim tunnel, measured under flow velocities used during AMR testing (post‐test).

#### 2.3.1 Standard metabolic rate

Although there is some debate as to the duration of starvation, acclimation and measurement times required to “truly” measure the best proxy for basal/resting metabolism (Chabot et al., 2016; Fry, 1971), we use the term SMR to retain consistency with that typically found in the literature pertaining to respirometry in fishes. Following 24‐hr fasting, fish were weighed, randomly assigned to a swim tunnel, and allowed to acclimate undisturbed for a minimum of 3 hr prior to the collection of data. During both the acclimation stage and SMR measurements, within‐tunnel velocity was maintained at 5 cm/s to allow dissolved oxygen to be uniformly distributed inside each tunnel—observations suggested that this rate of water flow did not provoke sustained swimming activity in fish. Automated intermittent (stop‐flow) respirometry cycles were conducted at consecutive 30‐min intervals consisting of three phases: A 7‐min tunnel flush period followed by tunnel closure to prohibit water/oxygen exchange, a 3‐min acclimation period, and a 20‐min measurement period. Stop‐flow cycles began ca. 2 hr prior to the dark phase of the photoperiod; however, data collection for calculation of SMR was restricted to ten measurement cycles in the absence of light stimulus, following “sunset” and preceding “sunrise (00:00–05:00).”

Metabolic rate was estimated based on the decline in oxygen concentration over the measurement period in each cycle, corrected for background respiration. Dissolved oxygen concentration inside each swim tunnel was measured at 1‐s intervals by fiber optic sensors, which transmitted signals to oxygen monitoring equipment and software (OXY‐4 & AutoResp; Loligo Systems, Denmark). Oxygen consumption was calculated as the slope of the linear regression of measured O_2_ concentration on time—all data were tested for linearity, and only measurements with a correlation ≥0.9 were used. To avoid bias due to background respiration (i.e., overestimation of an individual's respiration), tunnel‐specific pretest O_2_ consumption was subtracted from each measurement of O_2_ consumption.

We first tested for allometric differences among groups via analysis of covariance (ANCOVA) of log_10_ transformed data (absolute metabolic rate & total wet weight). Critically, results confirmed that the shape of the size–SMR relationship was similar among morphotypes (i.e., no significant difference in scaling exponents; *F*_[2]_ = 1.14, *p *=* *.323; Fig. S2a). Additionally, 95% confidence interval estimates of the common scaling exponent (0.72 ≤ b ≤ 0.99) suggested an isometric relationship with absolute SMR over the range of body sizes comprising the current dataset. This was further reinforced by contrasting models fit to morph‐specific data, wherein linear model fit was no different from that of a power curve (Fig. S2c,e,g). The absence of allometric differences allowed us to express data as a simplified mass‐specific metabolic rate (MO_2_; mg O_2_ kg^−1^ hr^−1^), thereby facilitating comparisons with other published estimates, and most critically, permitting subsequent tests for correlated selection between trait pairs. MO_2_ was calculated based on the following equation:

(2) $${\text{MO}}_{2}=\frac{\left({\left[O_{2}\right]}_{t0}-{\left[O_{2}\right]}_{t}\right)}{t}\times \frac{V}{W},$$

where [O_2_]_*t*0_−[O_2_]_*t*_ is the amount of oxygen consumed by fish (in mg O_2_/L), *t* is the time interval of measurement period (in s), *V* is the volume of the swim tunnel (in L), *W* is the body weight of the fish (in kg). The minimum value of mass‐specific oxygen consumption rate (MO_2 min_), obtained from ca. 10 trials, was used to estimate SMR.

#### 2.3.2 Active metabolic rate

Active metabolic rate was also estimated by intermittent respirometry; however, during the measurement phase, water velocity in each tunnel was set to 80% of an individual's *U*_crit_ to exclude activation of white muscle metabolism based on anaerobic respiration (Beddow & McKinley, 1999; Webb, 1971) and thereby potentially capture respiration at (or near) the peak of aerobic metabolism (Eliason & Farrell, 2016). While some have criticized the term AMR as being vague, favoring instead the term maximum metabolic rate (Norin & Clark, 2016), it bears reiteration that the original definition of AMR was explicitly based on respiration during peak/maximum aerobic activity (Fry, 1971). We note also that preliminary results from a larger (*n* = 200) companion study using BAR fish suggest that respiration at 80% of *U*_crit_ is similar to that observed following recovery from maximum sustained swimming (EPOC; Morozov, unpublished data), suggesting that we have effectively measured respiration at or near peak aerobic activity. Nevertheless, we favor retention of the original terminology to describe the parameter we measured (i.e., AMR) to distinguish it from unambiguous estimates of maximum metabolic rate. Whether our measure of AMR corresponds to the “true” measure of maximum aerobic respiration or is merely reflective of a proportion of it, however, is irrelevant to study objectives (i.e., inferring the nature of selection acting upon aspects of metabolic physiology).

One round of intermittent respirometry lasted for 15 min consisting of three periods: flush (7 min), acclimation (3 min), and measurement (5 min). During the flush phase, water velocity was decreased to 5 cm/s, which served as a recovery period for fish between AMR trials. Three AMR trials were conducted for each fish—oxygen consumption/slope corrections were based on post‐test measurements of background respiration—with AMR estimated from the trial with the maximum slope. Absolute data were analyzed as previously described for SMR: first assuring the absence of allometric differences among morphs (i.e., no significant difference in scaling exponents; *F*_[2]_ = 0.54, *p *=* *.585; Fig. S2b); secondly, inferring an isometric relationship with absolute AMR over the range of body sizes comprising the current dataset (0.58 ≤ b ≤ 1.02; Fig. S2d,f,h). Simplified mass‐specific oxygen consumption (MO_2max_) was also calculated as per Equation (2), serving as our final estimate of AMR in subsequent analyses.

### 2.4 Lateral plate coverage

Following respirometry, fish were euthanized by cervical dislocation, fixed in 10% buffered formalin for approximately 2 weeks, then transferred to 70% ETOH. Sample preparation involved a modification of Potthoff's (1984) protocol in which pigmentation was removed by bleaching in a solution of H_2_O_2_ and KOH, and lateral plates were stained with Alizarin red. The left side of every individual was photographed at a standard distance with a common reference scale placed next the fish. Measurements were taken from digital images using the image analysis software ImageJ (Abràmoff, Magalhães, & Ram, 2004). These included standard length (SL) and the area covered by nonstructural plates (i.e., the lateral plates not buttressing the dorsal and pelvic spines). Note that only the first 20 nonstructural plates were measured because of difficulties with the identification of small caudal plates, typically associated with the keel region—for a detailed description of lateral plate identification and numbering, see Bergstrom and Reimchen (2003).

### 2.5 Data analysis

#### 2.5.1 Comparison among populations/morphs

The R statistical computing language/software was used for all analyses (version 3.0.1; R Development Core Team, 2013). Replicate measurements for SMR, AMR, and *U*_crit_ were performed on a subset of individuals (*n* = 12) one week following initial measurements. To compare the first and the second measurements, we used a paired Welch's *t* test, which indicated no significant differences between measurements in any of the traits (*p* > .3).

To test for mean trait differences among populations, we used the Bayesian mixed‐effects modeling framework implemented in the “MCMCglmm” package (Hadfield, 2010). Population/morph was treated as a fixed effect—with effects/coefficients contrasted against the ancestral group (BAR)—and family was included as a random term. To test specifically for differences between freshwater populations, we ran a series of additional models using the same framework, but with data restricted to the KAR and PUL groups. Models were run with a burn‐in of 800,000 iterations, followed by an additional 200,000 iterations from which each 200th point of the Markov Chain was sampled to reduce autocorrelation of estimates. Significance of contrasts was evaluated via *p*‐values profiled from the sampling chain. Population‐specific estimates for each trait, conditioned on random effects, were based on the posterior mode and were bounded by 95% posterior density interval estimates (PDIs). We also explored bivariate relationships between all pair‐wise trait combinations within each population. We used “MCMCglmm” to model the linear relationship between each pair of traits, incorporating random variation among families. Models were run with an initial burn‐in of 50,000 iterations and a sampling chain of 50,000 iterations, from which each 50th estimate was retained. Significance (i.e. p‐Values) and model coefficients were profiled from the sampling chains.

#### 2.5.2 Testing for signatures of selection

To ascertain whether natural selection or random genetic drift was responsible for trait divergence, we employed a model of multivariate trait differentiation expected under neutrality (Ovaskainen, Karhunen, Zheng, Arias, & Merilä, 2011), as implemented in the package “driftsel” (Karhunen, Merilä, Leinonen, Cano, & Ovaskainen, 2013). In this model, differentiation from a common ancestral population expected under drift is derived from both neutral genetic markers and the pedigrees of experimental animals. To estimate population‐level coancestry, we used genotype data (available from Dryad; https://doi.org/10.5061/dryad.s6h18) from previously published work that included our study populations (Leinonen et al., 2012). First, we used the program “LOSITAN” to identify which markers were putatively under selection (Antao, Lopes, Lopes, Beja‐Pereira, & Luikart, 2008), retaining 19 putatively neutral loci for subsequent analyses (Table S1, *n* = 30 individuals per population). Next, we modeled neutral genetic differentiation among populations using an admixture model implemented in “RAFM” (Karhunen & Ovaskainen, 2012), a package integrated into the “driftsel” analytical pipeline. RAFM was run for a total of 300,000 iterations which included a burn‐in of 200,000 iterations, followed by 100,000 iterations from which each 50th position in the Markov chain was sampled. Estimates of divergence were checked for convergence and autocorrelation using the “CODA” package (Plummer, Best, Cowles, & Vines, 2006).

The next stage in the “driftsel” analytical pipeline involves incorporating the pedigree of experimental animals to estimate genetic covariance among traits and the degree of differentiation among populations in the absence of selection. We began with a fully parameterized model including all five traits (SMR, AMR, lateral plate area, *U*_burst_ and *U*_crit_):This was run for a burn‐in of 100,000 iterations followed by 10,000 iterations from which estimates were sampled from each 10th position in the Markov chain. Additionally, we sought to tease apart the likely targets driving potential correlated responses to selection by exploring divergence in all pair‐wise combinations of traits. These models required a shorter burn‐in to achieve convergence (15,000 iterations), but sampling chains (10,000 iterations) and thinning intervals (10) remained unchanged. Finally, we tested for evidence of directional selection on each unique trait (burn‐in and MCMC sampling as per bivariate models).

The signature of selection is reflected in the model parameter *S*: under neutral divergence the expected values of *S* is 0.5, whereas values <0.5 indicate stabilizing selection and those >0.5 reflect divergent selection (Ovaskainen et al., 2011). To determine the significance of *S*, we profiled 95% PDIs and *p*‐values from the posterior distribution of the 1,000 estimates derived from the sampling chain. Additionally, to better visualize differences between observed/contemporary phenotypic variation and that expected under drift, we modified the “viz.traits” plotting function in the “driftsel” package. The original function plots only confidence ellipses for the bivariate phenotypic distributions expected under drift from an ancestral population (A), and the centroids of contemporary phenotypes for the focal groups. Our modification includes plotting confidence ellipses around the centroid points to better visualize which populations overlap or diverge from the phenotypic distributions predicted under neutrality.

## 3 RESULTS

### 3.1 Mean differences among populations

#### 3.1.1 Metabolism

**Figure 2:**
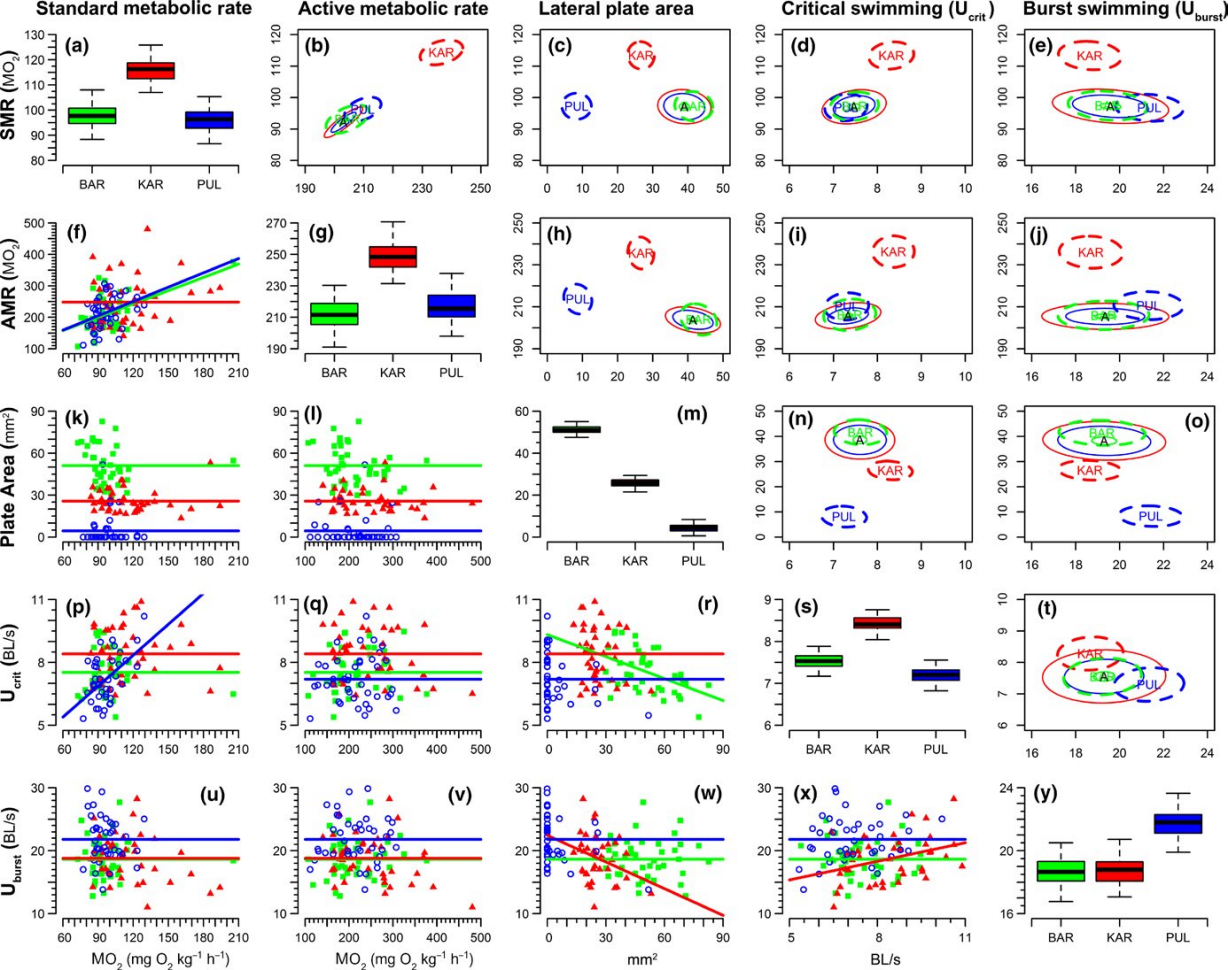
Interaction matrix for bivariate relationships among physiological, morphological, and swimming performance traits. Boxplots (on the diagonal) display mean differences among populations, conditioned on random effects. Scatter plots exhibiting contemporary/extant relationships between phenotypes are presented in the lower triangle. Nonsignificant relationships are indicated by horizontal lines at the conditioned mean of the ordinate phenotypic axis; nonhorizontal lines describe the slope of the relationship for significant correlations. The upper triangle displays results of bivariate tests of correlational selection. Thin, solid lines denote the phenotypic space expected to be occupied under neutral trait divergence from a theoretical ancestral (a) population; thick broken lines denote the parameter space observed in contemporary populations. 95% confidence ellipses are shown: overlapping solid and dashed ellipses, thus, denote neutral divergence whereas nonoverlapping pairs indicate a signature of correlational selection. In all panels marine/ancestral fish are plotted in green (BAR); low‐plated fish are in blue (PUL), and the small‐plated population (KAR) is in red

No significant differences in metabolic rate were found between the ancestral marine (BAR) and a “typical” freshwater population (PUL; Table 1). However, the small‐plated morph (KAR) demonstrated significantly higher SMR (Figure 2a) and AMR (Figure 2g) than both other populations. On average, SMR of the KAR population was 19% higher than in both BAR (Table 1, *p *=* *.008) and PUL populations (*p *=* *.020). In each population, AMR (Figure 2g) was roughly twofold greater than SMR. AMR of the small‐plated morph was 17% higher than the full‐plated morph (Table 1, *p *=* *.014) and 15% higher than that of the low‐plated morph (*p *=* *.030).

**Table 1:** Summary of mixed‐effects models. Coefficients describe differences of derived freshwater populations (Pop) from the mean of anadromous marine fish from the Barents Sea, the ancestral source of freshwater colonizers. Variance estimates correspond to random effects attributable to variation among family groups (*V*_Fam_) and residual model variance (*V*_resid_)

Trait	Fixed effects			Variance estimates	
	Pop	Coefficients (95% PDIs)	*p*‐Value		Posterior mode (95% PDIs)
SMR	KAR	18.60 (4.92 to 32.79)	.008	*V*_Fam_	106.94 (29.75 to 277.41)
	PUL	−1.35 (−15.07 to 12.42)	.840	*V*_resid_	292.25 (230.62 to 411.57)
AMR	KAR	36.78 (10.67 to 66.66)	.014	*V*_Fam_	1.53E‐02 (1.54E‐16 to 8.44E+01)
	PUL	3.98 (−25.26 to 32.00)	.794	*V*_resid_	3,847.46 (3,057.83 to 5,227.97)
Plate area	KAR	−25.27 (−30.54 to −19.41)	<.001	*V*_Fam_	5.82E‐02 (1.67E‐16 to 3.83E+01)
	PUL	−46.61 (−51.37 to −40.15)	<.001	*V*_resid_	109.58 (80.03 to 149.93)
*U*_crit_	KAR	0.88 (0.38 to 1.44)	.002	*V*_Fam_	1.89E‐05 (1.64E‐16 to 1.49E+01)
	PUL	−0.33 (−0.84 to 0.19)	.236	*V*_resid_	1.35 (0.97 to 1.67)
*U*_burst_	KAR	0.14 (−2.26 to 3.13)	.936	*V*_Fam_	3.71 (1.64 to 10.37)
	PUL	3.15 (0.69 to 6.09)	.030	*V*_resid_	7.37 (5.96 to 10.26)

#### 3.1.2 Lateral plate coverage

Second‐generation fish demonstrated the same plate architecture as their parental populations and could be clearly classified as full‐, small‐, and low‐plated morphs (Figure 2m). The number of plates in the BAR and KAR populations was identical, but the size of nonstructural lateral plates was considerably reduced in the small‐plated freshwater population (Figures 1c and 2m). In contrast, PUL fish largely lacked nonstructural lateral plates: 29 of 40 individuals did not have any nonstructural plates, eight had only the first posterior plate, and three had a partly covered posterior body (about 9–11 nonstructural plates). The greatest area of plate coverage of the posterior body was observed in the full‐plated morph, which corresponded to twice the area in small‐plated morph (Table 1, *p *<* *.001) and nearly 12 times greater plate area than that in the low‐plated morph (*p *<* *.001).

#### 3.1.3 Swimming performance

Small‐plated fish exhibited significantly higher critical swimming speeds than either marine fish (Table 1, *p *=* *.002) or the low‐plated morph (*p *<* *.001), with swimming speeds on average 12% and 17% greater, respectively. Conversely, low‐plated fish displayed significantly greater burst swimming speeds than either of their conspecifics. Their average speed was 17% and 14% higher than those from BAR (Table 1, *p *=* *.030) or KAR (*p *=* *.042), respectively. Although mean burst swimming speed did not differ between small‐ and full‐plated populations (Figure 2y; Table 1, *p* = .936), this may be complicated by a significant negative relationship between lateral plate area and burst speed in the KAR population (Figure 2w; *p *=* *.040).

### 3.2 Trait correlations

Measures of active and resting metabolism were positively correlated, but only for BAR (*p *<* *.001) & PUL (*p *=* *.032) populations (Figure 2f). Standard metabolic rate was also positively correlated with critical swimming speed in PUL (Figure 2p; *p *<* *.001), but not with burst swimming speed. No significant correlations were detected between active metabolic rate and the remaining traits in any population. Lateral plate coverage was negatively correlated with critical swimming speed in the full‐plated population (Figure 2r; *p *=* *.002), and with burst swimming speed in the small‐plated population (Figure 2w; *p *=* *.040). Burst swimming speed was also positively correlated with critical swimming speed in the small‐plated morph (Figure 2x; *p *=* *.030).

### 3.3 Signatures of selection

Lateral plate area exhibited a highly significant signal of directional selection shaping differences among populations/morphs (Table 2). Likewise, both AMR and SMR showed evidence for adaptive differentiation (Table 2). Conversely, neither *U*_crit_ nor *U*_burst_ differed from neutral expectation (Table 2).

**Table 2:** Signatures of selection (*S*) underlying differences in performance traits among three‐spined stickleback populations as obtained with “driftsel” package. Point estimates of *S* are based on the posterior mode, and are bound by 95% posterior density interval estimates (95%PDIs). Significance (*p*) is profiled from the posterior distribution of the estimates

Trait(s)	*S* (95% PDIs)	*p*‐Value
Univariate models of directional selection		
SMR	0.983 (0.932–1.000)	<.001
AMR	1.000 (0.999–1.000)	<.001
Plate area	1.000 (0.999–1.000)	<.001
*U*_crit_	0.605 (0.181–0.995)	.338
*U*_burst_	0.567 (0.138–0.988)	.390
Bivariate models of correlational selection		
SMR—AMR	1.000 (0.999–1.000)	<.001
SMR—Plate area	1.000 (0.999–1.000)	<.001
SMR—*U*_crit_	0.976 (0.895–1.000)	<.001
SMR—*U*_burst_	0.978 (0.908–1.000)	<.001
AMR—Plate area	1.000 (0.999–1.000)	<.001
AMR—*U*_crit_	1.000 (0.999–1.000)	<.001
AMR—*U*_burst_	1.000 (0.999–1.000)	<.001
Plate area—*U*_crit_	1.000 (0.999–1.000)	<.001
Plate area—*U*_burst_	1.000 (0.999–1.000)	<.001
*U*_crit_—*U*_burst_	0.630 (0.189–0.999)	.297

Active metabolic rate and SMR also showed evidence indicative of correlational selection (Table 2), but this appears to be unique to the small‐plated population (Figure 2b). Likewise, bivariate distributions suggest both *U*_crit_ and *U*_burst_ may have evolved as a correlated response to selection on SMR (Figure 2d,e) and AMR (Figure 2i,j) within the small‐plated population. Evidence of significant correlational selection between lateral plate coverage and metabolic rate was observed in both freshwater populations, but not in marine fish (Figure 2c,h). Similarly, both measures of swimming performance show patterns consistent with a correlated response to selection on lateral plate coverage in freshwater morphs (Figure 2n,o). Finally, no signatures of correlational selection between swimming traits were observed in any population (Figure 2t).

## 4 DISCUSSION

Although performance is expected to mediate fitness (sensu Arnold, 1983), we detected no direct signatures of selection on either metric of swimming performance, despite significant differences in conditioned means among populations (Figure 2s,y). Conversely, selection has clearly played a direct role in shaping the morphological and physiological differences among the focal stickleback populations. In comparison with typical pond and marine populations, the small‐plated morph has not only a unique architecture of lateral plates, but also higher metabolic rate and prolonged swimming performance. Surprisingly, the low‐ and full‐plated morphs were identical in all observed physiological and performance characteristics, excluding burst swimming speed. These results run counter to the available, yet limited, information about differences in metabolic rate and prolonged swimming performance among freshwater and marine populations of sticklebacks (Dalziel, Vines et al., 2012; Kitano et al., 2010; Taylor & McPhail, 1986; Tudorache et al., 2007). Here, we consider these contradictions, and further discuss which evolutionary processes appear to be driving population differentiation in novel freshwater environments, focusing particularly on evidence of directional selection on metabolic rate and a potential correlated response to selection on plate coverage observed in swimming performance. We focus the discussion of observed differences in SMR in light of the “context‐dependence” hypothesis, which posits that the fitness effects of variation in SMR differ between environments (Burton et al., 2011).

### 4.1 Adaptive divergence in freshwater

Given the preponderance of evidence linking lateral plate loss to increased fitness in freshwater (reviewed in Barrett, 2010), it is perhaps not surprising that lateral plate coverage observed in low‐plated individuals was significantly less than that expected under a model of neutral trait evolution (Table 1). Although less pronounced, this pattern was also observed in the small‐plated morph: the axes corresponding to variation in plate area in all bivariate plots separate neutral expectation from observed phenotypic variation (Figure 2c,h,n,o). Thus, we can conclude that reduction in lateral plate size may also be a feature of adaptation to the lacustrine environment, as previously hypothesized (Leinonen et al., 2012). The precise nature of the selective advantage conferred by reduced plate size remains unsolved; however, there is evidence to suggest a link to increased predator avoidance (Leinonen, unpublished data).

#### 4.1.1 The importance of physiology

While morphological divergence has been the major thrust of stickleback research over the past decade, cementing the species as an exceptional model in evolutionary ecology (Gibson, 2005), there is mounting evidence of the importance of physiology in its adaptation to freshwater (Dalziel, Ou, & Schulte, 2012; DeFaveri, Shikano, Shimada, Goto, & Merilä, 2011; Di Poi, Bélanger, Amyot, Rogers, & Aubin‐Horth, 2016; Kitano et al., 2010). Thus, it is perhaps not surprising that metabolic rate also appears to be under selection in this system. While previous work has documented genetically based differences in metabolism between marine and freshwater populations (Dalziel, Ou et al., 2012; Dalziel, Vines et al., 2012), results were interpreted in the context of relaxed selection pressures in freshwater relative to the marine environment, with selection being inferred on the basis of phenotypic similarity across multiple populations. Conversely, this study is the first to formally test for—and confirm—that metabolic differences between marine and freshwater populations are at least partially due to selection: both standard and active metabolic rates showed signals of divergence consistent with selection acting separately on each, but also potentially simultaneously via correlational selection.

Active metabolic rate plays an important role in physiological differentiation and adaptation because this parameter defines the upper limit of oxygen consumption during maximum aerobic swimming capacity of fish (Pitcher & Hart, 1983). AMR is also essential in its contribution to metabolic scope, a parameter hypothesized to be essential to fitness for ectotherms inhabiting thermally stressful environments (Kassahn, Crozier, Pörtner, & Caley, 2009; Pörtner et al., 2006). Yet despite its importance, it is a trait less commonly featured in evolutionary/functional ecology, certainly far less so than more easily measured traits such as routine or standard metabolic rate. Two previous studies have contrasted marine and low‐plated sticklebacks: one in which AMR was measured at several percentage values of *U*_crit_, including one similar to ours (75%; Tudorache et al., 2007); another at velocities in excess of *U*_crit_ (Dalziel, Vines et al., 2012). In both, AMR was significantly greater in marine fish. Conversely, we found that the small‐plated morph exhibited the highest levels of AMR, and that the AMR of low‐plated fish did not differ significantly from that of marine fish. This discrepancy might be explained by methodological differences between studies, but this is unlikely given the similarity of our method with that used by Tudorache et al. (2007). Alternatively, these conflicting observations may simply reflect the scarcity of observations from which general conclusions may be drawn, or even a lack of generality to be found among populations, hinting instead at context‐dependence. It is, however, noteworthy that the small‐plated population showed significantly higher AMR, and that selection has played a role in its divergence. One potential explanation for this might be reflected in the physical properties of the different waterbodies. Karilampi is a relatively shallow basin, which should in turn create a more thermally variable environment relative to either the ocean or the larger and deeper Pulmankijärvi. This is hypothesized to favor correlational selection between AMR and SMR to reduce variation in metabolic scope and optimize thermal sensitivity and energetic allocation (Careau, Gifford, & Biro, 2014). Results are indicative of correlational selection between these traits (Figure 2b); however, discerning between this and other potential selective pressures is beyond this study. Nevertheless, irrespective of the particular mechanism at play in this system, we would suggest that more studies in other contexts are warranted to empirically evaluate intraspecific variation of AMR and its fitness consequences.

Although measures of standard metabolic rate are more commonly featured in the literature, general trends with respect to freshwater colonization by sticklebacks are no more consistent. For example, some authors have observed no differences in SMR between freshwater and marine morphs (Dalziel, Vines et al., 2012), while others have reported higher SMR in full‐plated marine populations (Kitano et al., 2010; Tudorache et al., 2007). As in the case of AMR, no previous study has formally evaluated the role of selection in shaping differences in SMR between freshwater and marine sticklebacks. In the current system, the “typical” low‐plated population did not differ from their marine conspecifics, nor did their contemporary phenotypic distribution differ from that expected under neutral drift. Instead, selection appears to have acted only in driving increased SMR in the small‐plated morph. Negative directional selection gradients have been reported underlying reduced SMR via survival probability in an invasive population of garden snails (*Cornu aspersum*) in central Chile (Artacho & Nespolo, 2009). Yet when surveyed less than a decade later, stabilizing selection on SMR was inferred in the same population and at the same geographic location, in which mortality was low for snails originating from multiple climatic zones; negative selection gradients were inferred in a contrasting climactic zone (Bartheld et al., 2015). Similar patterns of interannual variation in selection gradients on basal metabolic rate have also been observed in wild blue tits, *Cyanistes caeruleus* (Nilsson & Nilsson, 2016). Likewise, conflicting inference of negative and positive selection gradients have been observed in the common lizard (*Zootoca vivipara*), depending on whether fitness is defined via fecundity or survival probability, respectively (Artacho, Saravia, Ferrandière, Perret, & Le Galliard, 2015), and in different life stages of the marine bryozoan, *Bugula neritina* (Pettersen, White, & Marshall, 2016). Together, these discrepancies speak to the context‐dependent nature of the relationship between SMR and fitness (Burton et al., 2011; Careau & Garland, 2012).

### 4.2 What is the “context” of population differentiation in SMR?

Traditionally, there were two opposite views on this question. According to the “increased intake hypothesis,” individuals with high relative SMR tend to be characterized by larger internal organs (Chappell, Garland, Robertson, & Saltzman, 2007; Steyermark, Miamen, Feghahati, & Lewno, 2005) and higher maximum metabolic rate (Biro & Stamps, 2010; Nilsson, 2002). As a result, they are able to acquire and assimilate more energy for fitness‐related processes such as reproduction, development, and fertility (Boratyński & Koteja, 2010; McNab, 1980). In contrast, the “compensation hypothesis” suggests that lower SMR enhances the fitness of an organism by making more energy available for growth, survival and reproduction due to low self‐maintenance costs (Deerenberg, Overkamp, Visser, & Daan, 1998; Larivée, Boutin, Speakman, McAdam, & Humphries, 2010). While both of these hypotheses seek to explain SMR differences among individuals, they ignore the role of environmental conditions in promoting metabolic rate differentiation among populations (e.g., temperature, the size of a habitat, the presence of predators). By studying the influence of environmental variation on the relationships between SMR and fitness, Burton et al. (2011) combined these two competing hypotheses into the “context‐dependent” hypothesis wherein both negative and positive directional selection on SMR are the result of different “self‐maintenance” strategies under different environmental pressures. Hence the conflicting results of previous studies, which showed empirical evidence of both positive and negative directional selections on SMR (e.g. Boratyński, Koskela, Mappes, & Schroderus, 2013), might present different sides of the same coin. One particular case of this hypothesis is observed in trade‐offs between stress resistance and performance, wherein high‐stress resistance is associated with low SMR (Álvarez, Cano, & Nicieza, 2006).

Our results are consistent with the “context‐dependent” hypothesis: the small‐plated freshwater population with higher SMR originates from a small pond with a relatively depauperate fish community compared to the other populations, and thus, likely lower predation pressures (Leinonen et al., 2012). Such conditions are also likely to increase the relative strength of intraspecific competition, and concomitantly favor increasing performance and SMR in a population (Handelsman et al., 2013; Reznick, Butler, & Rodd, 2001). In contrast, the marine population is certain to face a high predator pressure; likewise, piscine predators are known to inhabit the other freshwater population. In both cases, the amount of energy required for maintenance of basic biological functions is expected to be under strong directional selection: because high SMR would increase the need for increased nutritional input, concomitant increases in foraging would be tantamount to increased risk‐taking behavior, thereby reducing survival probability in the strong predator regime (Huntingford et al., 2010; Killen, Marras, & McKenzie, 2011; Mueller & Diamond, 2001). Similar “context‐dependent” patterns in forming an optimum SMR have been observed in several populations of Trinidadian guppy from high‐ and low‐predation environments (Handelsman et al., 2013), but see Fu, Fu, Yuan, and Cao (2015) for conflicting evidence in a cyprinid. Although results are consistent with expectations under the “context‐dependent” hypothesis, we stress that this is merely a post hoc interpretation. Our study was never intended to test this hypothesis, and so we lack data to formally test it, although we suggest this may be a fruitful avenue of exploration in similar systems.

### 4.3 Evolution of performance as a correlated response to selection

It is generally believed that increased capacity for sprint swimming and manoeuvrability is advantageous in structured habitats with ambush predators, as experienced by freshwater sticklebacks (Barrett, 2010; Leinonen et al., 2011; Reimchen, 1992). Indeed the observation that the low‐plated PUL population exhibited significantly higher burst swimming speeds than their source/ancestral population is a result consistent with other comparisons between freshwater and marine sticklebacks (Bergstrom, 2002; Hendry, Hudson, Walker, Räsänen, & Chapman, 2011; Taylor & McPhail, 1986). However, given that the ubiquity of the low‐plate morph in freshwater is frequently cited as evidence of its optimality in freshwater, and the relatively high and divergent selection pressures in this environment (Barrett, 2010; Colosimo et al., 2005), it was surprising that we detected no evidence of selection acting directly on this trait. Although we detected no signature of directional selection, we did detect a clear bivariate signal between burst speed and lateral plate area—this was particularly strong in the low‐plated population (Figure 2o).

Likewise, the observation of increased critical swimming speed in the small‐plated population was particularly unexpected. Marine sticklebacks are largely anadromous, and given that spawning migrations from distant marine environments to coastal and/or freshwater habitats require higher prolonged swimming performance, marine sticklebacks were expected to have a greater critical swimming capacity. Indeed, several studies have demonstrated that marine populations of three‐spined sticklebacks are excellent prolonged swimmers, outperforming isolated pond or stream‐resident populations (Dalziel, Vines et al., 2012; Taylor & McPhail, 1986; Tudorache et al., 2007). Conversely, Schaarschmidt and Jürss (2003) reported that one of two pond populations of low‐plated sticklebacks had the same critical swimming speed as a marine (putatively ancestral) population. Likewise, we detected no differences in prolonged swimming performance among low‐plated freshwater fish and their full‐plated marine conspecifics. These discrepancies may be explained by an association between prolonged swimming performance and fitness in environments characterized by low‐predation pressures and high intraspecific competition, where success in searching for food could be the main factor for survival and reproduction (Biro & Stamps, 2010). Although this description is highly congruous with the ecology of Karilampi, we detected no direct signatures of selection on *U*_crit_. Why and/or how the small‐plated morph shows significantly greater *U*_crit_ than the other morphs may relate to metabolism.

As with *U*_crit_, we observed no significant differences between low‐plated and marine fish in either metabolic parameter, whereas both standard and active metabolic rate levels in the small‐plated morph were significantly higher. We initially reasoned that higher metabolic capacity might explain the increased critical swimming speed in the small‐plated KAR population, as variation in *U*_crit_ can be mediated and/or constrained by the total amount of aerobic energy available to an animal (Dalziel, Vines et al., 2012). Yet neither metabolic rate variable was correlated with *U*_crit_—in actuality there was a significant correlation between SMR and critical speed (Figure 2p), but only in the low‐plated PUL population. Although these observations would argue against metabolic differences explaining patterns in critical swimming speed, the connection may lie not in contemporary relationships amongst parameters, but rather in the past action of selection.

Bivariate models suggest that a multivariate response to selection may explain differences in *U*_crit_ among populations. Given the absence of a univariate signal of selection, and bivariate projections demonstrating a clear separation on one axis and potential overlap with another, we suggest that *U*_crit_ has evolved as a correlated response to selection for increased metabolic rate. This is most pronounced in relation to AMR in the small‐plated KAR population (Figure 2i): differences in ellipses describing drift/neutral and contemporary phenotypic distributions are predominantly driven by metabolism; however, divergence of ellipses is also apparent along the axis corresponding to critical swimming speed (abscissa), more so than observed in relation with SMR (Figure 2d) or plate area (Figure 2n). This interpretation also aligns with the hypothesis that selection acts more directly on behavior and/or energetics than on performance traits (Careau & Garland, 2012), a notion lending further urgency to arguments for inclusion of physiology in studies of adaptive divergence.

## 5 CONCLUSIONS

To our knowledge, this study provides the first evidence of directional selection on active and standard metabolic rate in marine and freshwater stickleback populations. Additionally, we show that selection is responsible for the reduction in lateral plate coverage in a small‐plated stickleback population in Lapland. We also emphasize the potential for correlational selection acting on both metabolic traits, as well as correlated responses on swimming performance to selection on both morphology and metabolism. Individuals from the small‐plated freshwater population, which inhabit a small isolated basin, demonstrate significantly higher levels of metabolic rate and aerobic swimming performance than sticklebacks from more complex freshwater and marine environments. These results align with the “context‐dependent” hypothesis, where ecological pressure plays the key role in shaping an optimum level of SMR. Moreover, the same context may potentially be applied in explaining AMR and prolonged swimming speed differentiation in novel freshwater environments, although such an extension of the hypothesis needs further studies on the effects of predation regime and intraspecific competition on metabolism, behavior, and performance.

## CONFLICT OF INTEREST

None declared.

## AUTHORS’ CONTRIBUTIONS

All authors were involved with the conception of the study. T.L., R.J.S.M. and S.M. conducted the experiments. Data analysis and drafting of the original manuscript were performed by R.J.S.M. and S.M. All authors contributed critically to subsequent drafts, and gave final approval for publication.

## DATA ACCESSIBILITY

Data available from the Dryad Digital Repository: https://doi.org/10.5061/dryad.73kb7
