# Supplementary material for: Selection on the morphology–physiology‐performance nexus: Lessons from freshwater stickleback morphs
Source: Ecol Evol. 2017 Dec 22;8(2):1286–99. doi: 10.1002/ece3.3644 (PMC5773335; doi:10.1002/ece3.3644)
Supplement: Supplementary file 1 [file ECE3-8-1286-s001.pdf]

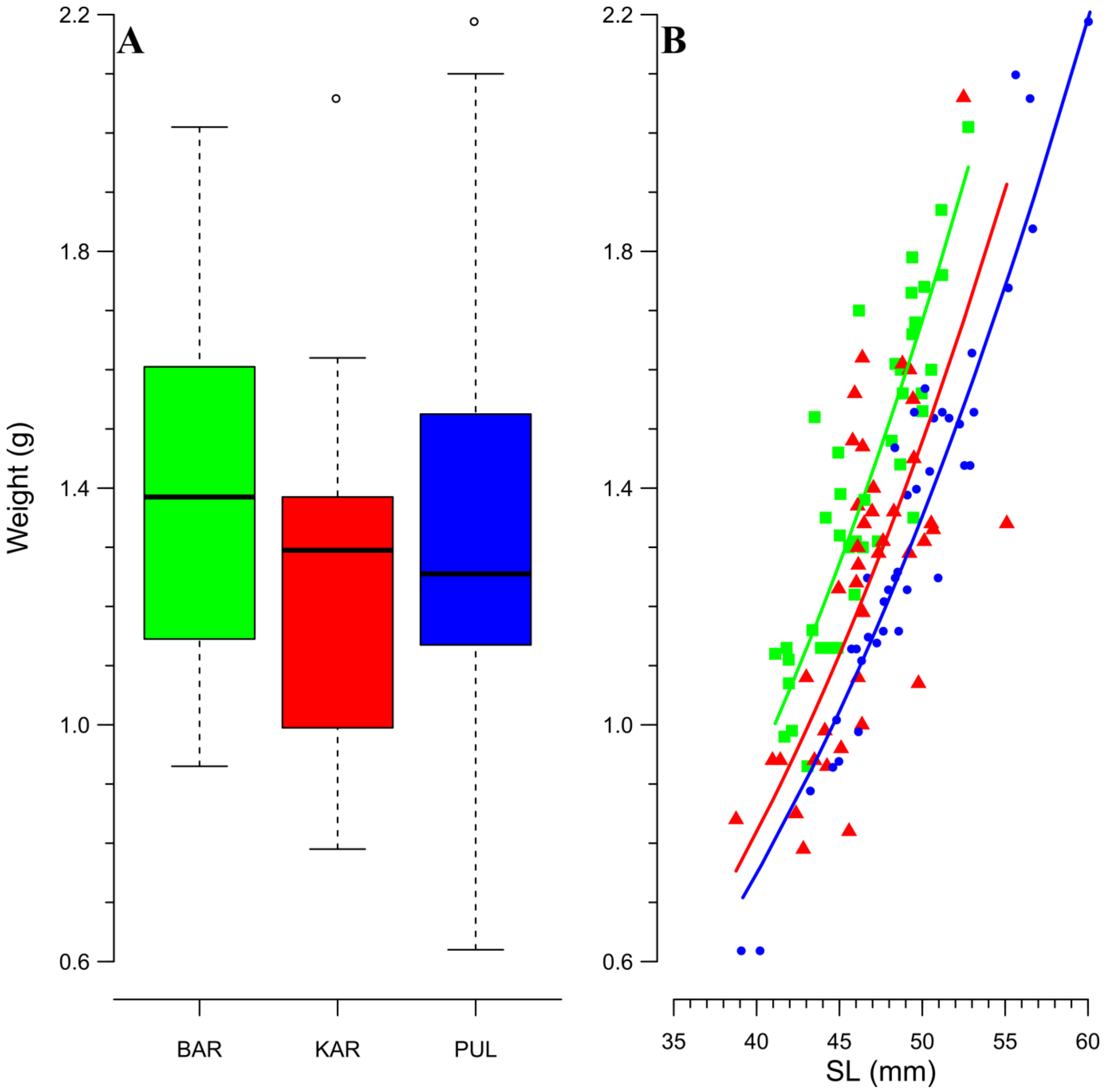

Figure S1 (A) Range of body mass variation for individuals from the respective stickleback populations used in this experiment. Boxes denote the interqartile range; whiskers denote approximate 95% confidence intervals. **(B)** Power curves describing the relationships between wet weight and standard body length for each population: intercepts of the models are different, but scaling exponents are similar.

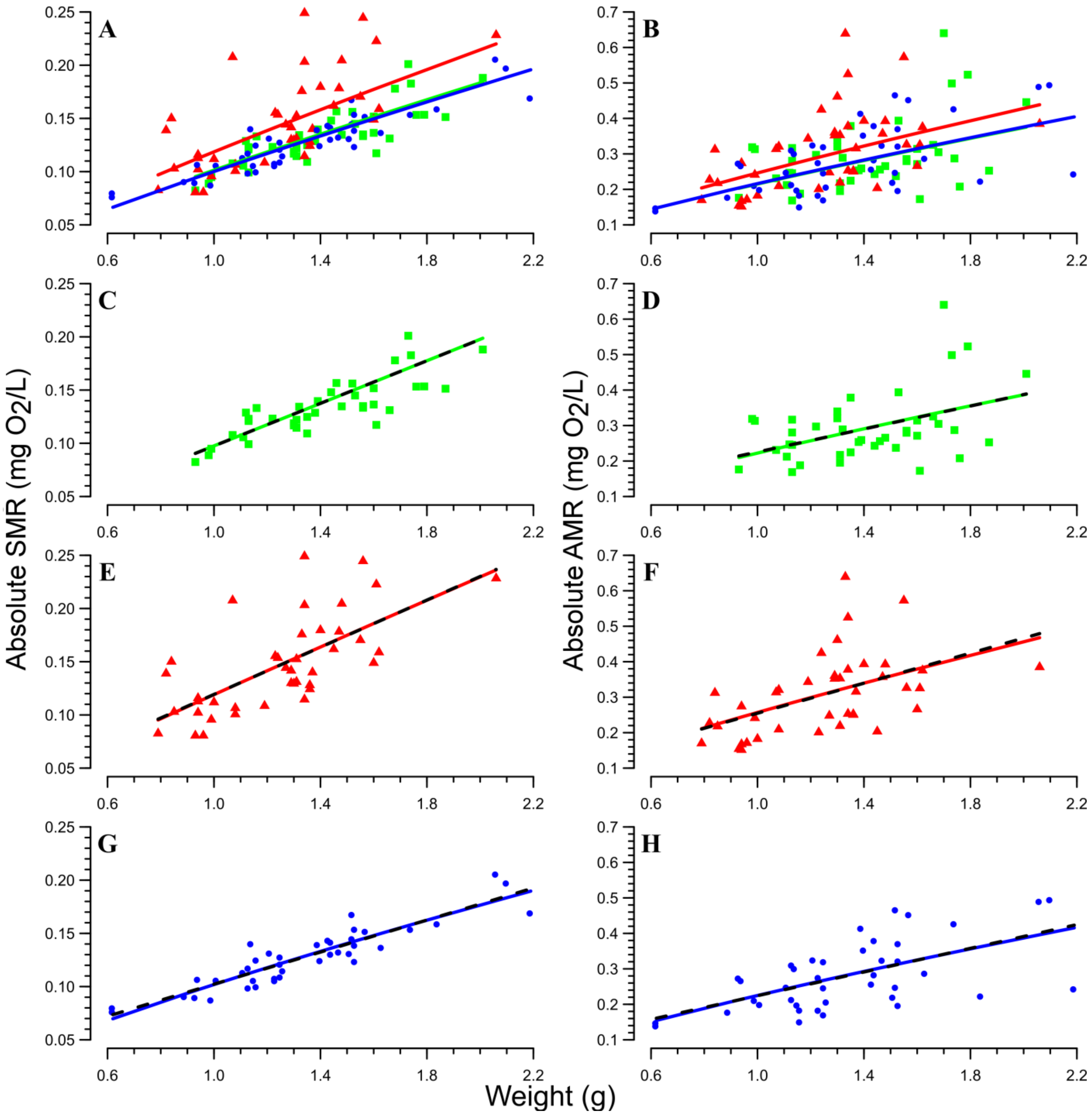

Figure S2 Bivariate relationships between weight and absolute metabolic rate: left panels display SMR, with corresponding AMR to the right. **(A & B)** Power curves corresponding to each population (green=BAR; red=KAR; blue=PUL). The intercepts corresponding to KAR are significantly greater than the other populations, but the scaling exponent is similar amongst all. **(C–H)** Visual comparison of power curves (solid line, colours as in A & B) and simple linear relationship (dashed black line) for each population.

Table S1. Loci used to model neutral divergence. Locus-specific estimates of allelic richness and F_{IS} were calculated using the '*hierfstat*' package in R. Estimates of average heterozygosity (Het) and F_{ST} were calculated using the program '*LOSITAN*' when testing for marker neutrality in among-population divergence.

Locus	No. Alleles			Allelic Richness			F_{IS}			Divergence	
	KAR	PUL	BAR	KAR	PUL	BAR	KAR	PUL	BAR	Het	F_{ST}
Gac1125	3	5	15	2.6	4.8	14.2	-0.033	-0.036	-0.069	0.834	0.181
Gac7033	2	5	9	1.9	4.1	7.7	-0.017	0.312	-0.030	0.802	0.467
Stn3	2	7	6	1.9	6.0	5.7	-0.017	0.030	0.131	0.900	0.461
Stn7	3	3	4	3.0	2.9	3.8	-0.055	0.010	0.199	0.743	0.347
Stn19	10	15	31	9.2	12.8	24.2	-0.071	-0.009	0.006	0.979	0.077
Stn34	4	24	23	3.9	18.7	17.1	0.119	0.033	-0.027	0.977	0.247
Stn38	1	2	3	1.0	2.0	2.9	–	-0.224	-0.095	0.266	0.102
Stn57	3	13	18	3.0	11.0	15.3	-0.270	0.046	-0.003	0.965	0.191
Stn76	1	3	5	1.0	3.0	4.9	–	-0.120	-0.138	0.745	0.467
Stn79	1	2	2	1.0	2.0	2.0	–	-0.036	0.140	0.166	0.130
Stn110	3	4	9	2.6	3.9	8.1	-0.045	0.008	0.006	0.894	0.283
Stn122	4	15	20	3.2	11.9	17.8	-0.082	-0.093	0.023	0.934	0.237
Stn132	2	8	10	2.0	7.1	9.3	0.364	-0.003	0.052	0.826	0.264
Stn135	1	5	6	1.0	4.6	6.0	–	-0.029	0.140	0.769	0.320
Stn163	4	11	14	3.6	8.5	11.1	0.139	-0.041	-0.019	0.932	0.330
Stn174	2	5	7	1.6	4.7	7.0	0.000	0.029	0.137	0.644	0.222
Stn195	2	8	8	2.0	7.2	7.9	-0.115	-0.054	-0.046	0.884	0.322
Stn211	1	5	8	1.0	5.0	7.6	–	0.079	0.154	0.924	0.470
Stn219	6	1	4	5.3	1.0	3.0	-0.009	–	-0.029	0.532	0.579
